# Adherence to antiretroviral treatment in adults with HIV: knowledge translation in website creation*

**DOI:** 10.1590/1518-8345.7196.4641

**Published:** 2025-08-18

**Authors:** Marcelo Ribeiro Primeira, Aiodelle dos Santos Machado, Tassiane Ferreira Langendorf, Daniel Gonzalo Eslava Albarracin, Cristiane Cardoso de Paula, Stela Maris de Mello Padoin

**Affiliations:** 1 Universidade Federal de Santa Maria, Santa Maria, RS, Brazil.; 2 Scholarship holder at the Coordenação de Aperfeiçoamento de Pessoal de Nível Superior (CAPES), Brazil.; 3 Fundaciòn Universitaria Caja de Compensación Familiar, Escola de Enfermagem, Bogotá, DC, Colombia.; 4 Universidade Federal de Santa Maria, Departamento de Enfermagem, Santa Maria, RS, Brazil.; 5 Scholarship holder at the Conselho Nacional de Desenvolvimento Científico e Tecnológico (CNPq), Brazil.

**Keywords:** HIV, Treatment Adherence and Compliance, Adult, Educational Technology, Translational Science, Biomedical, Nursing

## Abstract

to create a website to promote adherence to antiretroviral treatment for adults living with the human immunodeficiency virus.

methodological research in which the content was developed based on the synthesis of scientific evidence and validated by experts on the subject. The image content was created and linked to the text content for the development of the website. The analysis was performed based on the Content Validity Index by 20 experts recruited using the snowball technique.

the content was structured into three axes: self-efficacy, social support and quality of life. Each axis presents a concept, a total of 11 Life Situations with proposals for promoting adherence and 14 images. The overall Content Validity Index obtained a score >0.78. The website is freely accessible.

the educational-care technology typified as an informative website was validated by experts on the subject for use by the target population of adults living with the human immunodeficiency virus as a tool for promoting adherence to antiretroviral treatment.

## Highlights

**(1)** Care-educational technology for adherence to antiretroviral treatment for HIV. **(2)** Website with text and image content for adults living with HIV. **(3)** Health promotion using a non-pharmacological method and based on digital intervention. **(4)** Knowledge translation with health professionals and public policy managers.

## Introduction

The Joint United Nations Program (UNAIDS) leads the commitment and actions to prevent and control the epidemic of infection by the human immunodeficiency virus (HIV). This program, through guiding documents, directs the global response to prevent approximately 3.6 million HIV infections and 1.7 million deaths related to Acquired Immunodeficiency Syndrome (AIDS) by 2030^(1)^. This goal foresees the concentration of efforts to develop strategies for access to early diagnosis for 95% of people living with HIV. Of those who know their serological status, 95% are expected to be developing their treatment with antiretrovirals. And, of those (in treatment), 95% have suppressed viral load tests (undetectable)^(1)^.

The goal proposed by UNAIDS faces barriers to its achievement. Thus, a global systematic review sought to investigate factors associated with loss to follow-up among adults living with HIV^(2)^. This study, which included 20 countries in its analysis, identified that the most common factors were related to social and demographic issues such as age (adults aged 18 to 34), low education level (<9 years), economic problems, use of illicit drugs, stigma, lack of social support and distance between home and health unit. Clinical factors related to the start of antiretroviral use in the first six months and side effects are also highlighted. In addition, long waits for care, disorganization of health services and poor relationships between the health team and people living with HIV are factors associated with loss of follow-up of HIV treatment, compromising adherence^(2)^.

Therefore, it is understood that there is a direct implication with the need for care interventions to promote and maintain adherence to antiretroviral treatment, as well as local programs and national policies. Evidence synthesized in a systematic review presents effective interventions to improve self-management health outcomes. Most of the time, these interventions are used in combination, integrating skills training associated with telephone counseling, programs conducted by community health agents and technologies for symptom management. The benefits include increased self-efficacy, improved coping strategies, strengthened social support and improved quality of life (QoL)^(3)^.

In a Latin American context, the evaluation of the effectiveness of an educational intervention that aimed to increase the level of knowledge about the virus and promote adherence, developed in Mexico, showed a 90% increase in the level of knowledge among participants and a 70% increase in adherence to therapy. These results indicated that the educational intervention was effective for these outcomes^(4)^.

A Brazilian cohort study recommends that factors associated with non-adherence should be considered, including having good knowledge about treatment-related situations and not presenting symptoms that can impact the chances of adherence^(5)^. Therefore, the proposal is to translate knowledge about adherence to antiretroviral treatment from the synthesis of textual and image content, with a view to developing an educational resource to promote adherence to antiretroviral treatment among adults living with HIV. The objective of this study was to create a website to promote adherence to antiretroviral treatment among adults living with HIV.

## Method

### Study design

Methodological study for structuring and validating content production (text and images) and defining the type of technology. It was guided by the Canadian model of Knowledge Translation into Action, using the third phase of the creation cycle: knowledge product (third-generation study)^(6)^.

**Figure 1-:**
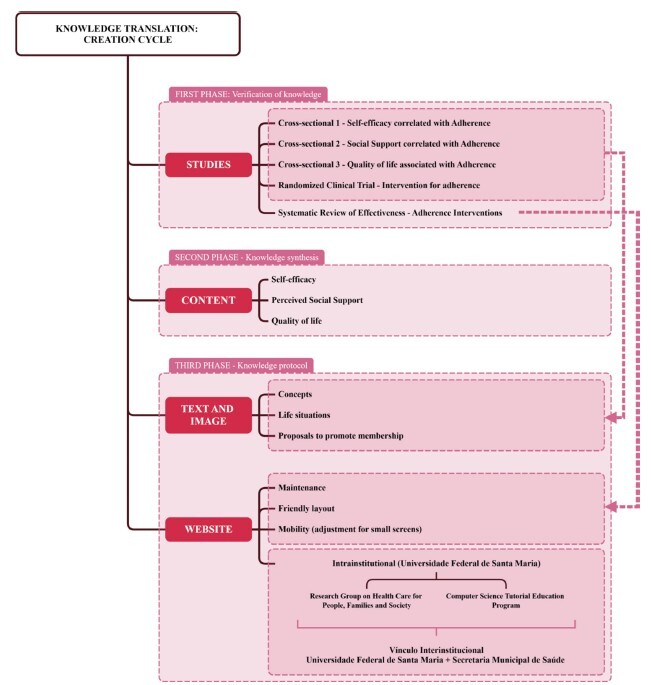
Knowledge Translation Organization Chart for promoting adherence to antiretroviral treatment for adults living with HIV. Santa Maria, RS, Brazil, 2024

The content structure was created based on the results of three cross-sectional studies^(7-9)^ and a randomized clinical trial (RCT)^(10)^ (first phase - knowledge verification/first-generation studies). The definition of the type of technology (third phase - knowledge product/third-generation study) for access to the developed content (second phase - knowledge synthesis/second-generation study) was established based on the results of a systematic review of the effectiveness of intervention for adherence to antiretroviral therapy for HIV in adults^(11)^ (Figure 1).

Therefore, the content was structured into three axes, anchored in theoretical frameworks: self-efficacy, social support and QoL. The concept of self-efficacy refers to the level of motivation and confidence in one’s ability to successfully develop behaviors, such as taking medications as prescribed^(12)^. Perceived social support is composed of emotional support, which presents the perception and satisfaction regarding the availability of listening, attention, information, esteem, company and emotional support in relation to HIV infection. It also encompasses the component related to instrumental support, which includes the perception and satisfaction regarding the availability of support in managing or resolving operational issues of treatment or health care, practical daily activities, material and/or financial assistance^(13)^.

As for QoL, the concept refers to the person’s perception of their position in life, considering their value systems, cultural context, as well as their goals, expectations, standards of living and concerns. Furthermore, the group of experts who created the concept of QoL concluded that its assessment should include different domains, which allows verification of the dimensions in which treatments will be effective, directing more appropriate therapeutic measures and possibly reducing health costs^(14)^.

Each axis was composed of the concept translated into language accessible to the target audience, being articulated with the creation of 11 Life Situations (LS) and propositions to promote adherence. Thus, self-efficacy led to the creation of seven LS and QoL of four LS related to coping with the chronicity of HIV infection in their daily lives. Social support guided the writing of the propositions to expand support for people living with HIV and to promote the outcome of adherence.

According to the textual content, images were created to represent the concepts and the LS. The creation of the images intended to illustrate the textual content corresponding to the concepts of the structuring axes and the LS so that the target audience would recognize themselves in the situations and propositions. To produce images, the team used the Google Meet platform, which offers audio and video connections for meetings. In the first meeting with the illustrator (who has a degree in Industrial Design with an emphasis on Visual Programming), the project team presented the images selected from the online graphic design platform Canva^®^. In the second meeting, the illustrator presented the sketches of the images and a color palette was decided that would demonstrate diversity. The third and fourth meetings were for critical review of the colored images and adjustments for the final version of the image content.

To define the type of technology, the summary of the systematic review was accessed^(11)^. In this summary, it was concluded that interventions combined with the use of mobile technologies with internet access have a potential for adherence of 12% more when compared to the standard service, which consisted of receiving identical exposure to introductory parts of the technology. In addition to not receiving information with interactive intervention components with visual and audio elements^(11)^.

Therefore, it was decided that the content would be made available for access through a website, considering the local context of creation of the knowledge product, with discussions with the Health Department of the municipality where the research team is based. The Higher Education Institution of the research team offered us the possibility of a multidisciplinary partnership with the Computer Science Tutorial Education Program for prototyping the website, which does not generate maintenance costs for the municipality, health policy and specialized service (implementation sites). In addition, the website can provide a user-friendly layout and be displayed in different screen sizes (mobile and desktop mode).

### Data collection period, participants and selection criteria

The validation of the textual content took place between August and October 2022. The participants were experts on the subject. The experts were selected based on their personal records on a platform for researchers’ CVs in Brazil (*Plataforma Lattes*), through an advanced search using the keywords HIV and AIDS. A spreadsheet was then created with the email addresses and telephone numbers available on the profiles of 105 researchers.

The snowball technique was also used to reach a varied sample of participants. Thus, out of the 22 participants, those who self-assessed with a score above three, based on criteria adapted from Fehring^(15)^, were considered experts. The adaptation in question referred to a doctorate and/or master’s degree with a thesis or dissertation, respectively, and/or specialization in the area, participation in research projects, publications in journals/events on the subject, and having worked in the area for at least one year. Two participants who did not obtain the minimum score defined were excluded. Thus, they were not included in the expert bank for calculating the Content Validity Index (CVI)^(16)^.

### Data collection, instruments used and study variables

The experts received a form divided into three sections by email. In the first, they answered self-assessment questions using the expert scoring system. In the second, they validated the textual content for relevance, clarity and pertinence, and in the third, the demographic data.

### Data processing and analysis

The CVI is a validity measure that measures the proportion or percentage of expert agreement on a given content^(16)^. Experts were asked to rate the relevance, clarity, and pertinence of each content provided in Google Forms. The concepts and LS were rated on a Likert-type scale considering the following options: 1 = Inadequate; 2 = Partially adequate; 3 = Adequate (requires minor changes); 4 = Fully adequate. To calculate the CVI for each content, the experts’ responses 3 and 4 were added together, dividing the result of this sum by the total number of responses obtained for the item. In order to interpret the CVI^(16)^, the result must be at least 0.78 to be considered acceptable^(16)^. CVI values should be used to guide decisions about reviews or rejections of validated content. If the CVI value is low, it may mean that the content does not have sufficient relevance, clarity, and pertinence^(16)^.

## Ethical aspects

This research followed the rules of Resolution No. 510/2016, which waives evaluation by the Research Ethics Committee because it is “[...] research that aims to deepen theoretical knowledge of situations that emerge spontaneously and contingently in professional practice, as long as they do not reveal data that can identify the subject [...]” and Resolution No. 674, of May 6, 2022, which reinforces this text. Thus, the link to the form containing the invitation to validate the content and explaining the exemption from evaluation by the Ethics Committee was sent to the experts. The data are archived and maintained under the responsibility of the researcher, in an electronic address and online file cloud specifically created for this research, with an electronic password for login considered strong by the company’s email provider/service Google^®^.

## Results

The website content was structured with the central theme of adherence and three axes: self-efficacy, social support and QoL, which influence the outcome of adherence to antiretroviral treatment. The translation of the textual content was based on the following summary of evidence: self-efficacy^(8)^ showed a significant correlation with overall adherence through experiences of annoyance, discrimination, rejection, insecurity in knowing how people (in close contact) would react upon learning of the diagnosis, nervousness, irritation, ingestion of large quantities of pills and other adverse effects caused by the medication. These were translated as LS. And they were articulated with social support^(7)^ and emotional support, which was significantly correlated with the domains of antecedents of adherence failure and doctor-patient communication. LS also comprised the QoL domains^(9)^, so that, among those positively associated with adherence, the following stood out: general function, concerns about the medication and trust in the health professional. The domain that negatively affected adherence was related to concerns about confidentiality. Regarding the RCT^(10)^, text messages based on social support were used to compose the guidelines that were linked to the LS.

The website content was validated by 20 experts, of whom 15 (78.9%) were female, the mean age was 41.89±10.954 years, 12 (57.9%) were trained nurses, 07 (31.6%) had master’s degrees (highest degree of qualification) and 13 (68.4%) worked in care (not exclusively). Among the experts, 11 (55.6%) worked in Rio Grande do Sul, Brazil. Table 1 shows the continuous variables and the CVI, which reached a score >0.78 in all the means of relevance, clarity and pertinence. As a result, the content for the website was validated by experts on the subject and, therefore, suitable for use in the technology and moving on to the next stage of evaluation with the target audience, that is, adults living with HIV.

**Table 1-:** Characterization of the sample of experts and Content Validity Index according to concepts and life situations (n = 20). Santa Maria, RS, Brazil, 2022

**Variable**	**Average**	**Minimum**	**Maximum**	**Standard deviation**
Fehring Model Score	6.26	4	13	2.621
Age	41.89	27	60	10.954
Time since Education	16.89	2	38	11.140
Time since Degree	6.74	1	35	7.578
Time since Professional Experience	15.526	2	35	10.895
**Content Validity Index**				
**Content**		**Relevance**	**Clarity**	**Pertinence**
**Concepts**				
Adherence		0.95	0.81	0.95
Self-efficacy		0.95	0.76	0.95
Social Support		0.95	0.67	0.86
Quality of life		1.00	0.86	1.00
**Life Situations**				
1		0.95	0.90	0.95
2		0.95	0.95	0.95
3		0.90	0.90	0.90
4		0.95	0.95	0.95
5		1.00	1.00	1.00
6		1.00	0.90	1.00
7		1.00	0.95	1.00
8		0.90	0.76	0.86
9		0.90	0.86	0.95
10		0.90	0.90	0.90
11		0.81	0.81	0.90
Average		**0.94**	**0.87**	**0.94**

**Figure 2-:**
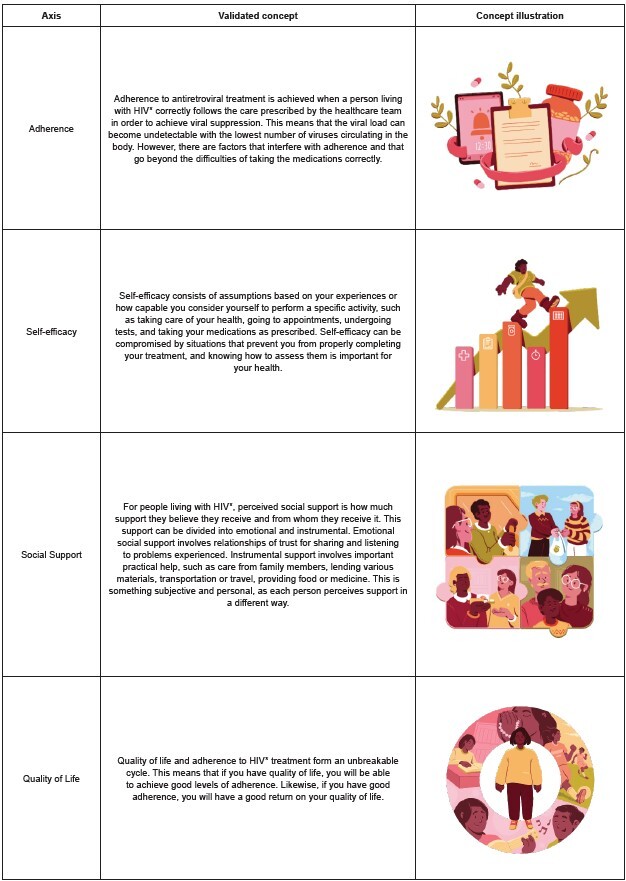
Validated concepts and final website illustrations. Santa Maria, RS, Brazil, 2022

The experts made suggestions that were considered by the research team and, when relevant, were added to the final version of the content. This stage involved the participation of a team qualified in language, grammar and communication. The final draft of the concepts, considering text and illustrations, is presented in Figure 2.

*HIV = Human Immunodeficiency Virus

**Figure 3-:**
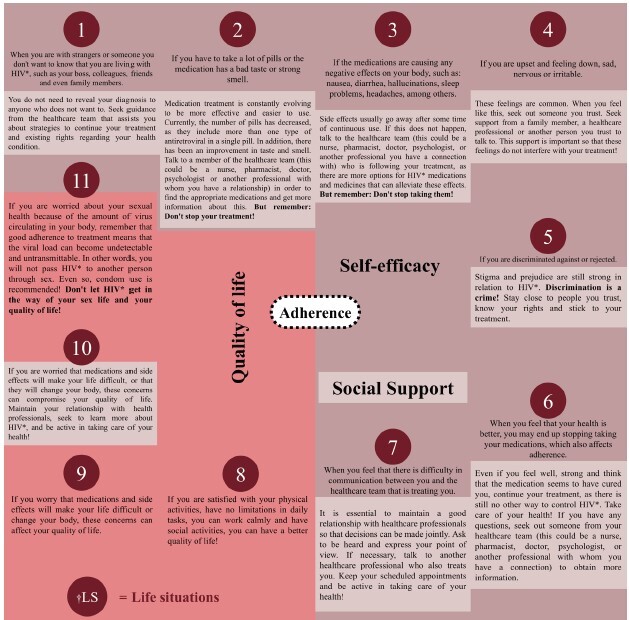
Representation of the articulation of the content of the ^†^LS with the structuring axes and the central theme – adherence. Santa Maria, RS, Brazil, 2022

In addition to the concepts, the validated textual content (Figure 3) gave rise to the other illustrations according to the LS.

*HIV = Human immunodeficiency virus; ^†^LS = Life situation

**Figure 4-:**
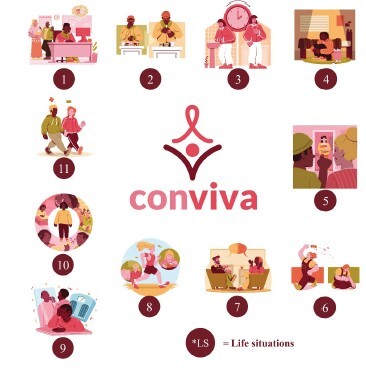
Visual identity and representative illustrations of Life Situations. Santa Maria, RS, Brazil, 2022

For the website’s visual identity, a logo was created with the word “*conviva*” (Figure 4), which conveys the idea of living and coping with HIV and the need to adapt to a new routine. For each LS, images were created to illustrate the textual content in order to engage the target audience and enhance understanding. In the experts’ assessment, the concept of QoL and LS 10 used the same illustration because they contain similar content (Figure 4). The website is available at the link: https://www.ufsm.br/pet/ciencia-da-computacao/conviva-1.

*LS = Life situation

## Discussion

The agreement rate among experts on the subject indicated that the website content is relevant, clear, and pertinent, and was validated by these participants for use with the target audience of adults living with HIV. The content of the VS is linked to the axes of self-efficacy, social support, and QoL, related to the outcome of adherence^(7-9)^, a central concept of this care-educational technology.

Studies that analyzed the barriers faced by adults living with HIV in relation to treatment adherence revealed, according to the results of a meta-analysis, that some barriers are individual, while others are related to health services^(17)^. Regarding the individual scope, the most frequent barriers were forgetting to take antiretrovirals and/or the effects of the medications^(18)^, being away from home, in the presence of strangers, and changes in daily routine^(19)^, and there was also the feeling of being well (asymptomatic) or sick^(5)^, depression^(20)^, and stigma^(21)^. It is worth noting that such barriers were proposed and translated in this study and comprise the textual and visual content of LS 1, 4, 5 and 6.

Additional barriers related to the treatment setting, which are programmatic in nature, include geographical distance to the healthcare facility and shortages of antiretroviral medications^(17)^. These barriers were not included in this proposal due to the complexity of the healthcare system and the varying local contexts.

Therefore, when such barriers are identified, it is necessary to propose strengthening the link to the service, seeking support and information in this person’s social assistance network in order to mitigate the implications for adherence to antiretroviral treatment. The fact that the website has the possibility of interfering in the retention of users in the services means that such people can seek help from the health team. One example concerns actions with the Social Service to ensure the rights of this population and public health policies related to HIV^(22)^.

All of the above indicates that combined interventions could ensure viral suppression through high levels of adherence to antiretroviral treatment^(17)^. Furthermore, adherence as a central concept of educational technology has been shown to be correlated with individual factors such as older age, higher resilience scores, and the perception of feeling sick^(23)^.

By articulating such evidence with the concept of self-efficacy to follow treatment^(19)^, the textual and visual content of this axis was created and validated, related to seven LS. As a determining factor for continuous treatment, the perception of self-efficacy allows people living with HIV to achieve good adherence results, without presenting clinical manifestations that, as the years go by, can interfere with their perception of health.

Likewise, social support, despite being a significant factor associated with good adherence results, is still insufficient to achieve success, since the overlapping of factors intervening in adherence must be considered^(24)^. Among them, in the dimension of social support, communication between the user and the health professional who cares for them stands out, as there is a statistical correlation with history of failure to adhere^(7)^. These issues are linked to the LS that involve clinical situations (LS 2, 3, 4, 6, 7, 9, 10 and 11) since, when the end user identifies with concerns related to situations of care for their health, it is assumed that they will be able to follow the guidelines based on adequate communication between the user and the health professional. This is because, among the factors of loss of follow-up of antiretroviral treatment, is the precarious relationship between the health team and people living with HIV^(2)^.

The articulation of the concepts of adherence to QoL is presented in four LS (LS 8 to 11). One study indicates that there is a feedback loop between QoL and adherence to HIV treatment, so that if a person living with HIV has good scores in the QoL assessment, they will be able to achieve good levels of adherence; in the same way, if such a person has good adherence, they will have a good return on their QoL^(9)^.

Given the fact that adherence and QoL represent a cycle, the assessment of QoL indicates that the environment in which the person lives can generate lower scores in this aspect^(24)^. This issue is related to LS1, in which the person does not reveal the diagnosis to strangers or acquaintances who they do not want to know about their serological status, which may be in the workplace or in the family. This LS is also contemplated and articulated with the concept of self-efficacy.

Likewise, the QoL dimension that assesses psychological health showed low scores compared to reference groups^(25)^. Low scores regarding psychological health reduce QoL and adherence. Therefore, the assessment of QoL provides adequate care regarding the dimensions of physical and psychological health, social relationships, environment, level of independence and spirituality of this population.

When comparing people who have not yet started treatment for HIV with antiretrovirals, those who were undergoing treatment reported significant improvements in physical^(23)^, emotional, mental health and in daily activities^(26)^. This comparison confirms that early diagnosis and treatment promotes QoL.

The results of a systematic review of qualitative studies, carried out in 2020, which aimed to explore the experiences and attitudes of people living with HIV, indicated that the moment of diagnosis is important due to the feelings that arise, such as: disappointment, sadness, fear, despair, lack of awareness and pain. Thus, health professionals should be prepared to support individuals during moments of vulnerability and to address different types of stigma: social, self-stigma and stigma from health professionals. Therefore, the social support of these professionals is highly valued and is linked to improving the QoL of these people^(27)^. The issue of stigma is addressed in LS1 and LS5.

Still in the field of stigma, the QoL of people living with HIV is impacted, particularly in the domain of sexual activity. An undetectable viral load was seen as transformative in the affective and sexual trajectories of women between 18 and 30 years old living with HIV^(28)^. When interviewing young people living with HIV/AIDS, a Brazilian study conducted in Salvador (BA) demonstrated that this population experiences the need to negotiate pleasure and prevention and position themselves as “risk” subjects. By adopting the notion of an undetectable viral load, they feel safe in prevention and transform feelings of fear, rejection and the “possibility” of “danger” for others (seronegative)^(28)^.

A study conducted in Sweden concluded that sexual satisfaction of women living with HIV is related to QoL^(29)^. In relation to people with penises, living with HIV can trigger erectile dysfunction, impairing this dimension of QoL^(30)^.

Despite advances in the biomedical field, social issues related to HIV, such as stigma and prejudice, still impede affective-sexual relationships. Stigma transforms the body of someone living with HIV into a potential transmitter of the virus, a body that poses a risk and is synonymous with promiscuity. Furthermore, stigma and prejudice promote feelings of guilt, which remain present in an active or latent form. Guilt arises because the person has become infected or has put themselves at risk. Furthermore, the feeling of responsibility persists not only for their infection, but also for the possibility of infecting another person with whom they may have a relationship, even with good adherence and an undetectable viral load^(31)^. The theme of sexuality and its connections with the concepts in this study translated for the target audience of adults with HIV are represented in LS10 and 11.

Regarding the classification as a care-educational technology^(32)^ in the form of an informative website, it is clear that the use of this educational resource meets the need for innovative products to be low-cost, as is the case with sending text messages via telephone. However, sending text messages comes up against technological limitations, such as sending texts with many characters and inserting pictures. When images are sent along with text messages, it is seen to be effective in improving adherence and satisfaction with services^(33)^.

With the possibility of accessing the internet through web browsing applications pre-installed on smartphones, known as browsers or web browsers, it is possible to browse websites or webpages that are very similar to native applications, both in terms of layout and functionalities presented. The advantages of accessing mobile technologies directly through the smartphone’s native browser are: there is no need for internal memory to install several applications, the possibility of accessing content from any device, and the ability to switch between screen sizes and operating systems, whether mobile or not^(34-35)^.

Finally, the textual and visual language of this product will contribute to health literacy practices, considering the assumption that abstractions, conceptualizations, and social and cultural behaviors are what give meaning to the use of languages, including those disseminated online. Thus, the production of meaning from the production of layouts for online platforms aims to support users in making informed decisions and choices^(36)^ and meets the need to create an identity with the end user, generate interactivity, and engagement in the use of information.

The limitations of the study concern the non-inclusion of people living with HIV in the process of creating the content of the educational-care technology at this time. For future studies, it is recommended that this target population be engaged.

As for the contributions of the study, which resulted in an informative website, we highlight the advancement of scientific knowledge for the health and nursing area related to the application of research in a knowledge translation model. And, especially, the availability of a product whose usability is anchored in educational-care technology. This modality can contribute to the autonomy of adults living with HIV, providing them with support regarding their multidimensional health condition and LS. It can qualify health care when used in health services, and can be mediated by care professionals.

## Conclusion

The informative website created provides scientific knowledge that has been translated and validated by experts in the field regarding its relevance, clarity and pertinence for use by adults living with HIV. The structure of the textual and visual content is anchored by fundamental concepts, such as self-efficacy, social support and quality of life, which are directly related to the experiences of these adults. These concepts play a crucial role in adherence to treatment, which is the main objective of this care-educational technology.
