# Supplementary material for: Adherence to antiretroviral treatment in adults with HIV: knowledge translation in website creation
Source: Rev Lat Am Enfermagem. 2025 Aug 18;33:e4641. doi: 10.1590/1518-8345.7196.4641 (PMC12364458; doi:10.1590/1518-8345.7196.4641)
Supplement: Translation

# Translation: Adesão ao tratamento antirretroviral de adultos com HIV: tradução do conhecimento na criação de *website* *

**DOI:** 10.1590/1518-8345.7196.4642

**Authors:** Marcelo Ribeiro Primeira, Aiodelle dos Santos Machado, Tassiane Ferreira Langendorf, Daniel Gonzalo Eslava Albarracin, Cristiane Cardoso de Paula, Stela Maris de Mello Padoin

**Affiliations:** 1 Universidade Federal de Santa Maria, Santa Maria, RS, Brasil.; 2 Bolsista da Coordenação de Aperfeiçoamento de Pessoal de Nível Superior (CAPES), Brasil.; 3 Fundaciòn Universitaria Caja de Compensación Familiar, Escola de Enfermagem, Bogotá, DC, Colômbia.; 4 Universidade Federal de Santa Maria, Departamento de Enfermagem, Santa Maria, RS, Brasil.; 5 Bolsista do Conselho Nacional de Desenvolvimento Científico e Tecnológico (CNPq), Brasil.

**Keywords:** HIV, Cooperação e Adesão ao Tratamento, Adulto, Tecnologia Educacional, Ciência Translacional Biomédica, Enfermagem

## Abstract

criar um *website* para a promoção da adesão ao tratamento antirretroviral de adultos que vivem com o vírus da imunodeficiência humana.

pesquisa metodológica na qual o conteúdo foi elaborado a partir da síntese de evidências científicas, sendo validado por especialistas na temática. O conteúdo imagético foi criado e articulado ao conteúdo textual para o desenvolvimento do *website*. A análise foi realizada com base no Índice de Validade de Conteúdo por 20 especialistas recrutados pela técnica bola de neve.

o conteúdo foi estruturado em três eixos: autoeficácia, suporte social e qualidade de vida. Cada eixo apresenta um conceito, num total de 11 Situações da Vida com proposições para promoção da adesão e 14 imagens. O Índice de Validade de Conteúdo global obteve pontuação >0,78. O *website* tem acesso livre.

a tecnologia cuidativo-educacional tipificada *website* informativo foi validada por especialistas na temática para uso pela população-alvo de adultos que vivem com vírus da imunodeficiência humana como ferramenta para promoção da adesão ao tratamento antirretroviral.

## Destaques

**(1)** Tecnologia cuidativo-educacional para adesão ao tratamento antirretroviral para o HIV. **(2)** *Website* com conteúdo textual e imagético para adultos que vivem com HIV. **(3)** Promoção da saúde por método não farmacológico e baseado em intervenção digital. **(4)** Tradução do conhecimento com profissionais da saúde e gestores de políticas públicas.

## Introdução

O Programa Conjunto das Nações Unidas sobre HIV/AIDS (UNAIDS) lidera o compromisso e as ações de prevenção e controle da epidemia de infecção pelo vírus da imunodeficiência humana (HIV). Por meio de documentos orientadores, o programa direciona a resposta global de que deverão ser evitadas cerca de 3,6 milhões de infecções pelo HIV e 1,7 milhão de mortes relacionadas à Síndrome da Imunodeficiência Adquirida (aids) até 2030^(1)^. Essa meta prevê a concentração de esforços para o desenvolvimento de estratégias para o acesso a um diagnóstico precoce para 95% das pessoas que vivem com HIV. Das que conhecem seu status sorológico, espera-se que 95% estejam desenvolvendo seu tratamento com antirretrovirais. E, dessas (em tratamento), espera-se que 95% estejam com os exames de carga viral suprimida (indetectável)^(1)^.

A meta proposta pelo UNAIDS enfrenta barreiras para sua concretização. Dessa forma, uma revisão sistemática global buscou investigar fatores associados à perda de seguimento entre adultos que vivem com HIV^(2)^. O estudo, que incluiu 20 países em sua análise, identificou que os fatores mais comuns foram relacionados a questões sociais e demográficas como idade (adultos de 18 a 34 anos), baixa escolaridade (<9 anos), problemas econômicos, uso de drogas ilícitas, estigma, falta de suporte social e distância entre o domicílio e a unidade de saúde. Fatores clínicos relacionados ao início do uso dos antirretrovirais nos primeiros seis meses e efeitos colaterais também são evidenciados. Além disso, a longa espera por atendimento, a desorganização dos serviços de saúde e o relacionamento precário entre a equipe de saúde e as pessoas que convivem com HIV são fatores associados à perda de seguimento do tratamento para o HIV comprometendo a adesão^(2)^.

Então, compreende-se que existe uma implicação direta com a necessidade de intervenções assistenciais para a promoção e manutenção da adesão ao tratamento antirretroviral, bem como programas locais e políticas nacionais. Evidências sintetizadas em revisão sistemática apresentam intervenções eficazes para melhorar os resultados de autogestão da saúde. Na maioria das vezes, essas intervenções são usadas de modo combinado, integrando o treinamento de habilidades associadas com aconselhamento por telefone, programas conduzidos por agentes comunitários de saúde e tecnologias para gestão dos sintomas. Os benefícios incluem o aumento da autoeficácia, aprimoramento das estratégias de enfrentamento, fortalecimento do suporte social e maior qualidade de vida (QV)^(3)^.

Em contexto latino-americano, a avaliação da efetividade de intervenção educacional, cujo objetivo era aumentar o nível de conhecimento acerca do vírus e promover a adesão, desenvolvida no México, apresentou 90% de aumento do nível de conhecimento entre os participantes e 70% de aumento da adesão à terapia. Esses resultados indicaram que a intervenção educacional foi efetiva para esses desfechos^(4)^.

Um estudo de coorte brasileiro recomenda que fatores associados à não-adesão devem ser considerados, entre os quais ter um bom conhecimento acerca de situações relacionadas ao tratamento bem como não apresentar sintomas podem impactar nas chances de adesão^(5)^. Então, tem-se a proposta de traduzir conhecimentos de adesão ao tratamento antirretroviral a partir da síntese do conteúdo textual e imagético, tendo em vista o desenvolvimento de um recurso educacional para a promoção da adesão ao tratamento antirretroviral de adultos que vivem com HIV. O objetivo deste estudo foi criar um *website* para a promoção da adesão ao tratamento antirretroviral de adultos que vivem com HIV.

## Método

### Delineamento do estudo

Estudo do tipo metodológico para a estruturação e validação do conteúdo textual, produção imagética e definição do tipo de tecnologia. Foi guiado pelo modelo canadense de Tradução do Conhecimento em Ação, sendo utilizada a terceira fase do ciclo de criação: produto do conhecimento (estudo de terceira geração)^(6)^.

A estruturação do conteúdo foi criada com base nos resultados de três pesquisas transversais^(7-9)^ e um ensaio clínico randomizado (ECR)^(10)^ (primeira fase - averiguação do conhecimento/estudos de primeira geração). A definição do tipo de tecnologia (terceira fase - produto do conhecimento/estudo de terceira geração) para acesso ao conteúdo desenvolvido (segunda fase - síntese do conhecimento/estudo de segunda geração), foi estabelecida a partir do resultado de uma revisão sistemática de efetividade de intervenção para a adesão à terapia antirretroviral para o HIV em adultos^(11)^ (Figura 1).

**Figura 1-:** Organograma da Tradução do Conhecimento para a promoção da adesão ao tratamento antirretroviral de adultos que vivem com HIV. Santa Maria, RS, Brasil, 2024

Logo, o conteúdo foi estruturado em três eixos, ancorados em referenciais teóricos: autoeficácia, suporte social e QV. O conceito de autoeficácia se refere ao nível de motivação e confiança nas capacidades pessoais de desenvolver determinados comportamentos com sucesso, como tomar medicamentos conforme a prescrição^(12)^. O suporte social percebido é composto pelo suporte emocional, que apresenta a percepção e satisfação quanto à disponibilidade de escuta, atenção, informação, estima, companhia e apoio emocional em relação à infecção pelo HIV. Também engloba o componente referente ao suporte instrumental, que contempla a percepção e satisfação quanto à disponibilidade de apoio no manejo ou resolução de questões operacionais do tratamento ou do cuidado de saúde, de atividades práticas do cotidiano, de ajuda material e/ou financeira^(13)^.

Quanto à QV, o conceito se refere à percepção da pessoa sobre a sua posição na vida, considerando seus sistemas de valores, contexto cultural, bem como seus objetivos, expectativas, padrões de vida e preocupações. Ainda, o grupo de especialistas que criou o conceito de QV concluiu que sua avaliação deve contemplar diferentes domínios, o que permite a verificação das dimensões nas quais os tratamentos serão efetivos, direcionando medidas terapêuticas mais apropriadas e possivelmente diminuindo os custos em saúde^(14)^.

Cada eixo foi composto pelo conceito traduzido em linguagem acessível para o público-alvo, sendo articulado com a criação de 11 Situações da Vida (SV) e proposições para promoção da adesão. Assim, a autoeficácia conduziu a criação de sete SV e a QV de quatro SV relacionadas com o enfrentamento da cronicidade da infecção pelo HIV no seu dia a dia. O suporte social guiou a redação das proposições para ampliar o apoio às pessoas que vivem com HIV e para a promoção do desfecho de adesão.

Segundo o conteúdo textual, foram criadas as imagens que representassem os conceitos e as SV. A elaboração das imagens pretendeu ilustrar o conteúdo textual correspondente aos conceitos dos eixos estruturantes e às SV de modo que o público-alvo se reconhecesse nas situações e proposições. Para produção imagética, a equipe utilizou a plataforma *Google Meet*, que oferece conexões de áudio e vídeo para as reuniões. No primeiro encontro com o ilustrador (o qual possui Graduação em Desenho Industrial com ênfase em Programação Visual), a equipe do projeto apresentou as imagens selecionadas da plataforma de *design* gráfico *onlineCanva* ^®^. No segundo, o ilustrador apresentou os croquis das imagens e foi decidida a paleta de cores que demonstrasse diversidade. O terceiro e quarto encontros foram para revisão crítica das imagens coloridas e ajustes para versão final do conteúdo imagético.

Para definição do tipo de tecnologia, foi acessada a síntese da revisão sistemática^(11)^. Nessa síntese, concluiu-se que as intervenções combinadas com o uso de tecnologias móveis com acesso à internet, apresentam potencial de adesão de 12% a mais se comparado com o atendimento padrão que foi receber exposição idêntica a partes introdutórias da tecnologia. E, não receber informações com componentes de intervenção interativa com elementos visuais e de áudio^(11)^.

Por isso, foi definido que o conteúdo seria disponibilizado para acesso por meio de um *website*, considerando o contexto local de criação do produto do conhecimento, com discussões com a Secretaria de Saúde do município sede da equipe de pesquisa. A Instituição de Ensino Superior da equipe de pesquisa em questão oferece a possibilidade de parceria multidisciplinar com o Programa de Educação Tutorial da Ciências da Computação para prototipação do *website*, o que não gera custos de manutenção para o município, política de saúde e serviço especializado (locais de implementação). Além da possibilidade de o *website* proporcionar *layout* amigável e ser exibido em diferentes tamanhos de tela (modo *mobile* e *desktop*).

### Período da coleta de dados, participantes e critérios de seleção

A validação do conteúdo textual ocorreu entre agosto e outubro de 2022. Os participantes foram especialistas no tema. Os especialistas foram selecionados levando em conta o registro pessoal em plataforma de currículos de pesquisadores no Brasil (Plataforma Lattes), por meio de busca avançada com as palavras-chave HIV e aids. Então, foi criada uma planilha com endereços eletrônicos e telefones disponíveis nos perfis de 105 pesquisadores.

Também, foi utilizada a técnica de bola de neve para o alcance de uma amostra variada de participantes. Dessa forma, dos 22 participantes, foram considerados especialistas aqueles que se autoavaliaram com pontuação acima de três, a partir de critérios adaptados de Fehring^(15)^. A adaptação em questão se referiu ao doutorado e/ou mestrado com tese ou dissertação, respectivamente, e/ou Especialização na área, a participação em projetos de pesquisa, o fato de possuir publicações em periódicos/anais de eventos na temática e ter tempo de atuação na área de no mínimo um ano. Foram excluídos dois participantes que não obtiveram a pontuação mínima definida, os quais não foram inseridos no banco de especialistas para cálculo do Índice de Validade de Conteúdo (IVC)^(16)^.

### Coleta de dados, instrumentos utilizados e variáveis do estudo

Os especialistas receberam, por *e-mail*, um formulário dividido em três seções. Na primeira, responderam a questões de autoavaliação no sistema de pontuação de especialistas. Na segunda, validaram o conteúdo textual quanto à relevância, clareza e pertinência e, na terceira, os dados demográficos.

### Tratamento e análise dos dados

O IVC é uma medida de validade que mede a proporção ou a porcentagem de concordância de especialistas sobre determinado conteúdo^(16)^. Foi solicitado aos especialistas que classificassem a relevância, clareza e pertinência de cada conteúdo disposto no *Google Forms*. Os conceitos e SV foram classificados a partir de uma escala tipo Likert considerando as seguintes opções: 1 = Inadequado; 2 = Parcialmente adequado; 3 = Adequado (necessita de pequenas alterações); 4 = Totalmente adequado. Para calcular o IVC de cada conteúdo, foram somadas as respostas 3 e 4 dos especialistas, dividindo o resultado dessa soma pelo número total de respostas obtidas para o item. Para interpretação do IVC^(16)^, o resultado deve ser de, no mínimo, 0,78, para ser considerado aceitável^(16)^. Os valores de IVC devem ser utilizados para orientar decisões acerca de revisões ou rejeições dos conteúdos validados. Se o valor do IVC for baixo, pode significar que o conteúdo não apresenta relevância, clareza e pertinência suficientes^(16)^.

## Aspectos éticos

Esta pesquisa seguiu as normas da Resolução n. 510/2016, que dispensa avaliação no Comitê de Ética em Pesquisa por se tratar de “[...] pesquisa que objetiva o aprofundamento teórico de situações que emergem espontânea e contingencialmente na prática profissional, desde que não revelem dados que possam identificar o sujeito [...]” e da Resolução n. 674, de 06 de maio de 2022, que reforça este texto. Assim, enviou-se, para os especialistas, o *link* do formulário em que continha o convite para a validação do conteúdo, explicitando a dispensa da avaliação no Comitê de Ética. Os dados estão arquivados e mantidos sob a responsabilidade da pesquisadora, em endereço eletrônico e nuvem de arquivos *online* especificamente criadas para esta pesquisa, possuindo senha eletrônica para *login* considerada forte pelo provedor/serviço de *e-mail* da empresa *Google* ^®^.

## Resultados

O conteúdo do *website* foi estruturado com o tema central referente à adesão e três eixos: autoeficácia, suporte social e QV, os quais influenciam o desfecho da adesão ao tratamento antirretroviral. Para tradução do conteúdo textual, sustentou-se na seguinte síntese de evidências: a autoeficácia^(8)^ apresentou correlação significativa com a adesão global por meio das experiências de aborrecimento, discriminação, rejeição, insegurança em saber como as pessoas (de convívio próximo) iriam reagir ao saber do diagnóstico, nervosismo, irritação, ingestão de grande quantidade de comprimidos e outros efeitos adversos causados pela medicação. Essas foram traduzidas como SV. E foram articuladas ao suporte social^(7)^ e ao suporte emocional que esteve correlacionado significativamente com os domínios de antecedentes de falha de adesão e comunicação médico-paciente. Também compuseram as SV os domínios da QV^(9)^, de modo que, dentre aqueles associados positivamente à adesão, houve destaque para: função geral, preocupações com a medicação e confiança no profissional de saúde. Já o domínio que interferiu de forma negativa na adesão relacionou-se à preocupação com o sigilo. Quanto ao ECR^(10)^, foram utilizadas as mensagens de texto, baseadas em suporte social para compor as orientações que foram vinculadas às SV.

O conteúdo do *website* foi validado por 20 especialistas, dos quais 15 (78,9%) eram do sexo feminino, a média de idade foi de 41,89±10,954 anos, 12 (57,9%) eram enfermeiros de formação, 07 (31,6%) eram mestres (maior grau de titulação) e 13 (68,4%) atuavam na assistência (não exclusivamente). Dentre os especialistas, 11 (55,6%) exerciam sua profissão no Rio Grande do Sul, Brasil. A Tabela 1 retrata as variáveis contínuas e o IVC, que atingiu a pontuação >0,78 em todas as médias de relevância, clareza e pertinência. Em função disso, o conteúdo para o *website* foi validado por especialistas no tema e, portanto, apto a compor a tecnologia e seguir para a próxima etapa de avaliação com público-alvo, ou seja, pessoas adultas vivendo com HIV.

**Tabela 1-:** Caracterização da amostra de especialistas e Índice de Validade de Conteúdo segundo conceitos e situações da vida (n = 20). Santa Maria, RS, Brasil, 2022

**Variável**	**Média**	**Mínimo**	**Máximo**	**Desvio-padrão**
Pontuação Modelo Fehring	6,26	4	13	2,621
Idade	41,89	27	60	10,954
Tempo de Formação	16,89	2	38	11,140
Tempo de Titulação	6,74	1	35	7,578
Tempo de Experiencia Profissional	15,526	2	35	10,895
**Indice de Validade de Conteúdo**				
**Conteúdo**		**Relevância**	**Clareza**	**Pertinência**
**Conceitos**				
Adesão		0,95	0,81	0,95
Autoeficácia		0,95	0,76	0,95
Suporte Social		0,95	0,67	0,86
Qualidade de Vida		1,00	0,86	1,00
**Situações da Vida**				
1		0,95	0,90	0,95
2		0,95	0,95	0,95
3		0,90	0,90	0,90
4		0,95	0,95	0,95
5		1,00	1,00	1,00
6		1,00	0,90	1,00
7		1,00	0,95	1,00
8		0,90	0,76	0,86
9		0,90	0,86	0,95
10		0,90	0,90	0,90
11		0,81	0,81	0,90
**Média**		**0,94**	**0,87**	**0,94**

Os especialistas emitiram sugestões que foram apreciadas pela esquipe de pesquisa e, quando pertinentes, foram acrescentadas na versão final da redação do conteúdo. Essa etapa contou com a participação de equipe qualificada quanto à linguagem, gramática e comunicação. A redação final dos conceitos, considerando texto e ilustrações, está apresentada na Figura 2.

**Figura 2-:** Conceitos validados e ilustrações finais do *website*. Santa Maria, RS, Brasil, 2022

*HIV = Vírus da Imunodeficiência Humana

Além dos conceitos, o conteúdo textual validado (Figura 3) deu origem às demais ilustrações conforme as SV.

**Figura 3-:** Representação da articulação do conteúdo das SV^†^ com os eixos estruturantes e o tema central – adesão. Santa Maria, RS, Brasil, 2022

*HIV = Vírus da imunodeficiência humana; ^†^SV = Situação da vida

Para a identidade visual do *website,* foi criado logotipo com a palavra “conviva” (Figura 4), que comporta a ideia de viver e convier com o HIV e a necessidade de se adaptar a uma nova rotina de vida. Para cada SV, foram criadas imagens para ilustrar o conteúdo textual de modo a engajar o público-alvo e potencializar a compreensão. Na avaliação dos especialistas o conceito de QV e a SV 10 utilizaram a mesma ilustração por conter conteúdos afins (Figura 4). O *website* está disponível no link: https://www.ufsm.br/pet/ciencia-da-computacao/conviva-1.

**Figura 4-:** Identidade visual e ilustrações representativas das Situações da Vida. Santa Maria, RS, Brasil, 2022

*SV = Situação da vida

## Discussão

O índice de concordância entre os especialistas na temática indicou que o conteúdo do *website* apresenta relevância, clareza e pertinência sendo validado por tais participantes para uso com o público-alvo de adultos que vivem com HIV. O conteúdo das SV está articulado aos eixos autoeficácia, suporte social e QV, relacionados com o desfecho da adesão^(7-9)^, conceito central dessa tecnologia cuidativo-educacional.

Estudos que analisaram as barreiras enfrentadas por adultos que vivem com HIV para aderir ao tratamento revelaram, de acordo com resultados de uma metanálise, que algumas barreiras são de âmbito individual e outras relacionadas aos serviços de saúde^(17)^. No que tange ao âmbito individual, encontra-se, com maior frequência, o esquecimento da ingestão dos antirretrovirais e/ou os efeitos dos medicamentos^(18)^, o fato de estar fora de casa, na presença de pessoas estranhas e a mudança na rotina diária^(19)^, e, ainda, havia o sentimento de estar bem (assintomático) ou doente^(5)^, a depressão^(20)^, e o estigma^(21)^. Destaca-se que tais barreiras foram propostas e traduzidas neste estudo e compõem o conteúdo textual e imagético das SV 1, 4, 5 e 6.

Existem outras barreiras relacionadas ao local de realização do tratamento, as quais são de cunho programático. Dentre elas, cita-se a distância geográfica até o serviço de saúde e quanto ao estoque de antirretrovirais em falta^(17)^. Essas barreiras não foram apresentadas nesta proposta devido à complexidade relacionada com o sistema de saúde e distintos contextos locais.

Então, quando tais barreiras são identificadas, faz-se necessário propor o fortalecimento do vínculo ao serviço, buscar apoio e informações nas redes sociais da pessoa, de modo a amenizar as implicações para a adesão ao tratamento antirretroviral. O fato de o *website* ter a possibilidade de interferir na retenção dos usuários aos serviços quer dizer que tais pessoas podem buscar auxílio na equipe de saúde. Um exemplo diz respeito a ações junto ao Serviço Social para a viabilização dos direitos desse segmento da população e das políticas públicas de saúde relacionadas ao HIV^(22)^.

Tudo o que foi exposto indica que intervenções combinadas poderiam garantir supressão viral por meio de níveis elevados de adesão ao tratamento antirretroviral^(17)^. Além disso, a adesão como conceito central da tecnologia cuidativo-educacional tem se mostrado correlacionada com fatores individuais como a faixa etária elevada, escores de resiliência mais altos e a percepção de sentir-se doente^(23)^.

Ao articular tais evidências com o conceito de autoeficácia para seguir o tratamento^(19)^, foram criados e validados o conteúdo textual e imagético deste eixo, relacionados a sete SV. Como um fator determinante para o tratamento contínuo, a percepção de autoeficácia permite que a pessoa que vive com HIV alcance bons resultados de adesão, não apresentando manifestações clínicas que, na medida que passam os anos, podem interferir em sua percepção de saúde.

Da mesma forma, o suporte/apoio social, mesmo sendo um fator significativo associado aos bons resultados de adesão, ainda é insuficiente para se obter o sucesso, pois deve-se considerar a sobreposição de fatores intervenientes na adesão^(24)^. Entre eles, destaca-se, na dimensão do suporte social, a comunicação entre a pessoa usuária e profissional de saúde que a atende, pois evidencia-se a correlação estatística com antecedentes de falha de adesão^(7)^. Tais questões estão articuladas com as SV que envolvem as situações clínicas (SV 2, 3, 4, 6, 7, 9, 10 e 11) uma vez que, quando o usuário final se identificar com preocupações relacionadas com situações de cuidados com sua saúde, pressupõe-se que poderá atender as orientações a partir de comunicação adequada entre usuário e profissional de saúde. Isso porque, dentre os fatores de perda de seguimento do tratamento antirretroviral, está o relacionamento precário entre a equipe de saúde e as pessoas que vivem com HIV^(2)^.

A articulação dos conceitos de adesão ao de QV está apresentada em quatro SV (SV8 a 11). Um estudo aponta que há um ciclo que se retroalimenta entre a QV e a adesão ao tratamento para o HIV, de modo que, se a pessoa que vive com HIV tem bons escores na avaliação da QV, poderá alcançar bons níveis de adesão; da mesma forma, se tal pessoa está com boa adesão, terá um bom retorno na sua QV^(9)^.

Diante do fato da adesão e QV representarem um ciclo, a avaliação da QV aponta que o ambiente no qual a pessoa vive pode gerar menores escores nesse aspecto^(24)^. Tal questão está relacionada à SV1 em que a pessoa não revela o diagnóstico para pessoas estranhas ou conhecidas que ela não quer que saibam de sua condição sorológica, pode ser no ambiente de trabalho ou na família. Essa SV também está contemplada e articulada com o conceito de autoeficácia.

Da mesma forma, a dimensão da QV que avalia a saúde psicológica apontou escores baixos em comparação com grupos de referência^(25)^. Escores baixos quanto à saúde psicológica diminuem a QV e a adesão. Portanto, a avaliação da QV proporciona cuidado adequado quanto às dimensões da saúde física, psicológica, relações sociais, meio ambiente, nível de independência e espiritualidade dessa população.

Ao comparar pessoas que ainda não iniciaram o tratamento para o HIV com antirretrovirais, aqueles que estavam em tratamento relataram melhoras significativas na saúde física^(23)^, emocional, mental e nas atividades diárias^(26)^. Essa comparação confirma que o diagnóstico e o tratamento precoces é promotor de QV.

Os resultados de uma revisão sistemática de estudos qualitativos, realizada em 2020, que objetivou explorar as experiências e atitudes de pessoas que vivem com HIV, apontou o momento do diagnóstico como importante devido aos sentimentos que se originam como: decepção, tristeza, medo, desespero, falta de consciência e dor. Dessa forma, os profissionais da saúde deverão se preparar tanto para tal momento de vulnerabilidade quanto para experiências com diferentes tipos de estigma: social, auto estigma e estigma dos profissionais de saúde. Portanto, o apoio social desses profissionais é bastante valorizado e está vinculado à melhoria da QV dessas pessoas^(27)^. A questão do estigma está abordada nas SV1 e SV5.

Ainda no campo do estigma, a QV de pessoas que vivem com HIV é impactada, particularmente, no domínio de atividade sexual. A carga viral indetectável foi vista como transformadora nas trajetórias afetivas e sexuais de mulheres entre 18 e 30 anos vivendo com HIV^(28)^. Ao realizar entrevistas com jovens vivendo com HIV/aids, um estudo brasileiro conduzido em Salvador (BA) demonstrou que essa população vivencia a necessidade de negociar o prazer e a prevenção e se posicionam como sujeitos de “risco”. Ao adotarem a noção de carga viral indetectável, sentem-se seguras na prevenção e transformam os sentimentos de medo, rejeição e a “possibilidade” de “perigo” para o outro (soronegativo)^(28)^.

Um estudo realizado na Suécia concluiu que a satisfação sexual de mulheres vivendo com HIV está relacionada à QV^(29)^. Em relação às pessoas com pênis, viver com HIV pode desencadear disfunção erétil, prejudicando essa dimensão da QV^(30)^.

Apesar dos avanços na área biomédica, as questões sociais relacionadas ao HIV, como estigma e preconceito, ainda são impeditivas de relacionamentos afetivo-sexuais. O estigma transforma o corpo de quem vive com a doença em um potencial transmissor do vírus, um corpo que oferece risco e é sinônimo de promiscuidade. Além disso, o estigma e o preconceito promovem o sentimento de culpa, que permanece presente de forma sempre ativa, ou de forma latente. A culpa surge pelo fato da pessoa ter se infectado, ou de ter se colado em risco. Ainda, perdura o sentimento de responsabilidade não somente por sua infecção, mas também da possibilidade de infectar outra pessoa com a qual venha a se relacionar, mesmo apresentando boa adesão e carga viral indetectável^(31)^. O tema da sexualidade e seus nexos com os conceitos neste estudo, traduzidos para o público-alvo de adultos com HIV estão representados nas SV10 e 11.

No que se refere à tipificação como uma tecnologia cuidativo-educacional^(32)^ na modalidade de *website* informativo, tem-se que o uso desse recurso educacional vai ao encontro da necessidade de produtos de inovação serem de baixo custo, assim como o envio de mensagem de texto por meio de telefone. No entanto, o envio de mensagens de texto esbarra nas limitações tecnológicas, quanto ao envio de textos com muitos caracteres e a inserção de figuras. Quando há o envio de imagens junto da mensagem de texto, percebe-se eficácia na melhoria da adesão e satisfação com serviços^(33)^.

Com a possibilidade de acessar a internet por meio de aplicativos de navegação na *web*, pré-instalados nos *smartphones*, conhecidos como *browser* ou *web browser*, é possível navegar por sites ou *webpages* muito semelhantes a aplicativos nativos tanto pelo *layout* quanto por funcionalidades apresentadas. As vantagens de acessar tecnologias móveis diretamente pelo navegador nativo do *smartphone* são: não haver necessidade de memória interna para instalar vários aplicativos, possibilidade de acessar o conteúdo de qualquer aparelho e a alternância entre tamanhos de tela e sistemas operacionais, sejam eles *mobiles* ou não^(34-35)^.

Por fim, a linguagem textual e imagética deste produto irá contribuir com as práticas de letramento em saúde, considerando o pressuposto de que as abstrações, conceitualizações e comportamentos sociais e culturais é que dão sentido ao uso das linguagens, incluindo aquelas divulgadas de modo *online*. Assim, a produção de sentido a partir da produção de *layouts* para plataformas *online* tem a intenção de apoiar os usuários na tomada de decisão e escolhas acertadas^(36)^ e vão ao encontro da necessidade de criar uma identidade com o usuário final, gerar interatividade e engajamento no uso das informações.

As limitações do estudo dizem respeito à não inclusão de pessoas vivendo com HIV no processo de criação do conteúdo da tecnologia cuidativo-educacional na presente oportunidade. Para estudos futuros, recomenda-se o engajamento de tal população-alvo.

Quanto às contribuições do estudo, que teve como produto um *website* informativo, destaca-se o avanço do conhecimento científico para a área de saúde e enfermagem relacionado à aplicação de pesquisa em um modelo de tradução do conhecimento. E, especialmente, pela disponibilidade de um produto que tem sua usabilidade ancorada em tecnologia cuidativo-educacional. Essa modalidade pode contribuir para a autonomia de adultos que vivem com HIV, fornecendo-lhes suporte quanto à sua condição multidimensional de saúde e SV. Poderá qualificar a atenção à saúde ao ser utilizada nos serviços de saúde, podendo ter a mediação de profissionais da assistência.

## Conclusão

O *website* informativo criado disponibiliza conhecimentos científicos que foram traduzidos e validados por especialistas no tema quanto à sua relevância, clareza e pertinência para ser utilizado por adultos vivendo com HIV. A estrutura do conteúdo textual e visual ancora-se em conceitos fundamentais, tais como autoeficácia, suporte social e qualidade de vida, que são diretamente relacionados às experiências desses adultos. Esses conceitos desempenham um papel crucial na adesão ao tratamento, que é o principal objetivo desta tecnologia cuidativo-educacional.

# Translation: Adhesión al tratamiento antirretroviral de adultos con VIH: traducción del conocimiento en la creación de un sitio *web* *

**DOI:** 10.1590/1518-8345.7196.4640

**Authors:** Marcelo Ribeiro Primeira, Aiodelle dos Santos Machado, Tassiane Ferreira Langendorf, Daniel Gonzalo Eslava Albarracin, Cristiane Cardoso de Paula, Stela Maris de Mello Padoin

**Affiliations:** 1 Universidade Federal de Santa Maria, Santa Maria, RS, Brasil.; 2 Becario de la Coordenação de Aperfeiçoamento de Pessoal de Nível Superior (CAPES), Brasil.; 3 Fundaciòn Universitaria Caja de Compensación Familiar, Escola de Enfermagem, Bogotá, DC, Colombia.; 4 Universidade Federal de Santa Maria, Departamento de Enfermagem, Santa Maria, RS, Brasil.; 5 Becaria del Conselho Nacional de Desenvolvimento Científico e Tecnológico (CNPq), Brasil.

**Keywords:** VIH, Cumplimiento y Adherencia al Tratamiento, Adulto, Tecnología Educacional, Ciencia Traslacional Biomédica, Enfermería

## Abstract

crear un sitio *web* para promover la adhesión al tratamiento antirretroviral en adultos que viven con el virus de la inmunodeficiencia humana.

investigación metodológica en la cual el contenido fue elaborado a partir de la síntesis de evidencias científicas y validado por especialistas en la temática. El contenido imagético fue creado y articulado al contenido textual para el desarrollo del sitio *web*. El análisis se realizó con base en el Índice de Validez de Contenido, calculado por 20 especialistas reclutados mediante la técnica de bola de nieve.

el contenido fue estructurado en tres ejes: autoeficacia, apoyo social y calidad de vida. Cada eje presenta un concepto, con un total de 11 Situaciones de la Vida con proposiciones para promover la adhesión y 14 imágenes. El Índice de Validez de Contenido global obtuvo una puntuación >0,78. El sitio *web* tiene acceso libre.

la tecnología cuidadivo-educativa tipificada como sitio *web* informativo fue validada por especialistas en la temática para su uso por parte de la población objetivo de adultos que viven con el virus de la inmunodeficiencia humana como herramienta para promover la adhesión al tratamiento antirretroviral.

## Destacados

**(1)** Tecnología cuidadivo-educativa para la adhesión al tratamiento antirretroviral del VIH. **(2)** Sitio *web* con contenido textual e imagético dirigido a adultos que viven con VIH. **(3)** Promoción de la salud mediante un método no farmacológico y basado en intervención digital. **(4)** Traducción del conocimiento con profesionales de la salud y gestores de políticas públicas.

## Introducción

El Programa Conjunto de las Naciones Unidas (UNAIDS) lidera el compromiso y las acciones de prevención y control de la epidemia de infección por el virus de la inmunodeficiencia humana (VIH). Este programa, por medio de documentos orientadores, dirige la respuesta global con el objetivo de evitar aproximadamente 3,6 millones de nuevas infecciones por el VIH y 1,7 millones de muertes relacionadas con el Síndrome de Inmunodeficiencia Adquirida (sida) hasta el año 2030^(1)^. Esta meta prevé la concentración de esfuerzos en el desarrollo de estrategias para garantizar el diagnóstico precoz en el 95 % de las personas que viven con VIH. De estas, se espera que el 95 % esté en tratamiento con antirretrovirales y, de estas (en tratamiento), que el 95 % tenga la carga viral suprimida (indetectable)^(1)^.

La meta propuesta por UNAIDS enfrenta barreras para su realización. En este sentido, una revisión sistemática global buscó investigar los factores asociados a la pérdida de seguimiento entre adultos que viven con VIH^(2)^. Este estudio, que incluyó a 20 países en su análisis, identificó que los factores más comunes estaban relacionados con cuestiones sociales y demográficas, tales como edad (adultos de 18 a 34 años), bajo nivel educativo (<9 años), problemas económicos, consumo de drogas ilícitas, estigma, falta de apoyo social y distancia entre el domicilio y el centro de salud. También se identificaron factores clínicos relacionados con el inicio del uso de antirretrovirales en los primeros seis meses y los efectos secundarios. Además, la larga espera por atención, la desorganización de los servicios de salud y la relación precaria entre el equipo de salud y las personas que viven con VIH fueron señaladas como causas de la pérdida de seguimiento del tratamiento, lo cual compromete la adhesión^(2)^.

De este modo, se comprende que existe una implicación directa con la necesidad de intervenciones asistenciales para promover y mantener la adhesión al tratamiento antirretroviral, así como programas locales y políticas nacionales. Evidencias sintetizadas en una revisión sistemática presentan intervenciones eficaces para mejorar los resultados de autogestión de la salud. La mayoría de las veces, estas intervenciones se utilizan de forma combinada, integrando el entrenamiento de habilidades con asesoramiento telefónico, programas dirigidos por agentes comunitarios de salud y tecnologías para el manejo de los síntomas. Los beneficios incluyen el aumento de la autoeficacia, la mejora de las estrategias de afrontamiento, el fortalecimiento del apoyo social y una mayor calidad de vida (CV)^(3)^.

En el contexto latinoamericano, la evaluación de la efectividad de una intervención educativa desarrollada en México –cuyo objetivo era aumentar el nivel de conocimiento sobre el virus y promover la adhesión– mostró un aumento del 90 % en el nivel de conocimiento entre los participantes y un incremento del 70 % en la adhesión a la terapia. Estos resultados indicaron que la intervención educativa fue efectiva para dichos desenlaces^(4)^.

Un estudio de cohorte brasileño recomienda considerar los factores asociados con la no adhesión, entre ellos, tener un buen conocimiento sobre situaciones relacionadas con el tratamiento y no presentar síntomas, lo cual puede impactar en las probabilidades de adhesión^(5)^. Así, se propone traducir conocimientos sobre la adhesión al tratamiento antirretroviral a partir de la síntesis del contenido textual e imagético, con el fin de desarrollar un recurso educativo para promover la adhesión al tratamiento antirretroviral en adultos que viven con VIH. El objetivo de este estudio fue crear un sitio *web* para la promoción de la adhesión al tratamiento antirretroviral en adultos que viven con VIH.

## Método

### Diseño del estudio

Estudio de tipo metodológico para la estructuración y validación del contenido textual, producción imagética y definición del tipo de tecnología. Fue guiado por el modelo canadiense de Traducción del Conocimiento en Acción, utilizándose la tercera fase del ciclo de creación: producto del conocimiento (estudio de tercera generación)^(6)^.

La estructuración del contenido fue elaborada con base en los resultados de tres investigaciones transversales^(7-9)^ y un ensayo clínico aleatorizado (ECA)^(10)^ (primera fase – averiguación del conocimiento/estudios de primera generación). La definición del tipo de tecnología (tercera fase – producto del conocimiento/estudio de tercera generación) para el acceso al contenido desarrollado (segunda fase – síntesis del conocimiento/estudio de segunda generación) fue establecida a partir del resultado de una revisión sistemática sobre la efectividad de intervenciones para la adhesión a la terapia antirretroviral en adultos con VIH^(11)^ (Figura 1).

**Figura 1-:** Organigrama de la Traducción del Conocimiento para la promoción de la adhesión al tratamiento antirretroviral en adultos que viven con VIH. Santa Maria, RS, Brasil, 2024

Así, el contenido fue estructurado en tres ejes, anclados en referentes teóricos: autoeficacia, apoyo social y CV. El concepto de autoeficacia se refiere al nivel de motivación y confianza en las propias capacidades para desarrollar con éxito conductas, como tomar los medicamentos según la prescripción^(12)^. El apoyo social percibido está compuesto por apoyo emocional, que abarca la percepción y satisfacción respecto a la disponibilidad de escucha, atención, información, estima, compañía y apoyo emocional frente a la infección por VIH. También incluye el componente relacionado con el apoyo instrumental, que contempla la percepción y satisfacción en cuanto a la disponibilidad de ayuda para manejar o resolver cuestiones operativas del tratamiento o del cuidado de la salud, de actividades prácticas cotidianas y de asistencia material y/o financiera^(13)^.

En cuanto a la CV, el concepto hace referencia a la percepción de la persona sobre su posición en la vida, considerando sus sistemas de valores, contexto cultural, así como sus objetivos, expectativas, patrones de vida y preocupaciones. Además, el grupo de especialistas que creó el concepto de CV concluyó que su evaluación debe contemplar distintos dominios, lo que permite verificar las dimensiones en las que los tratamientos serán efectivos, orientando medidas terapéuticas más apropiadas y posiblemente disminuyendo los costos en salud^(14)^.

Cada eje fue compuesto por el concepto traducido a un lenguaje accesible para el público objetivo, articulado con la creación de 11 Situaciones de la Vida (SV) y proposiciones para promover la adhesión. Así, la autoeficacia condujo a la creación de siete SV y la calidad de vida de cuatro SV, relacionadas con el afrontamiento de la cronicidad de la infección por VIH en la vida cotidiana. El apoyo social guió la redacción de proposiciones destinadas a ampliar el respaldo a las personas que viven con VIH y a promover el resultado de adhesión.

A partir del contenido textual se crearon imágenes que representaran los conceptos y las SV. La elaboración de las imágenes tuvo como objetivo ilustrar el contenido textual correspondiente a los conceptos de los ejes estructurantes y a las SV, de forma que el público objetivo se reconociera en las situaciones y proposiciones. Para la producción imagética, el equipo utilizó la plataforma *Google Meet*, que ofrece conexiones de audio y video para reuniones. En el primer encuentro con el ilustrador (quien posee un Grado en Diseño Industrial con énfasis en Programación Visual), el equipo del proyecto presentó las imágenes seleccionadas de la plataforma de diseño gráfico en línea *Canva* ^®^. En el segundo encuentro, el ilustrador presentó los bocetos de las imágenes y se decidió la paleta de colores que representara la diversidad. El tercer y cuarto encuentros fueron destinados a la revisión crítica de las imágenes a color y a los ajustes para la versión final del contenido imagético.

Para la definición del tipo de tecnología, se accedió a la síntesis de la revisión sistemática^(11)^. En dicha síntesis se concluyó que las intervenciones combinadas con el uso de tecnologías móviles con acceso a internet presentan un potencial de aumento en la adhesión del 12% en comparación con la atención estándar, que consistía en la exposición idéntica a partes introductorias de la tecnología y en no recibir información con componentes de intervención interactiva con elementos visuales y de audio^(11)^.

Por ello, se definió que el contenido sería puesto a disposición mediante un sitio *web*, considerando el contexto local de creación del producto de conocimiento, en diálogo con la Secretaría de Salud del municipio sede del equipo de investigación. La institución de educación superior a la que pertenece el equipo ofrece la posibilidad de una colaboración multidisciplinaria con el Programa de Educación Tutorial de Ciencias de la Computación para la prototipación del sitio *web*, lo que no genera costos de mantenimiento para el municipio, la política de salud ni el servicio especializado (lugares de implementación). Además, el sitio *web* permite un diseño amigable y puede visualizarse en distintos tamaños de pantalla (modo móvil y escritorio).

### Período de recolección de datos, participantes y criterios de selección

La validación del contenido textual se llevó a cabo entre agosto y octubre de 2022. Los participantes fueron especialistas en la temática. La selección de los especialistas se realizó a partir del registro personal en la plataforma brasileña de currículos de investigadores (*Plataforma Lattes*), mediante búsqueda avanzada con las palabras clave VIH y sida. Se creó una hoja de cálculo con direcciones de correo electrónico y teléfonos disponibles en los perfiles de 105 investigadores.

También se utilizó la técnica de bola de nieve para alcanzar una muestra variada de participantes. De los 22 participantes, se consideraron especialistas aquellos que se autoevaluaron con una puntuación superior a tres, según criterios adaptados de Fehring^(15)^. La adaptación se refirió a poseer doctorado y/o maestría con tesis o disertación, respectivamente, y/o especialización en el área, participación en proyectos de investigación, publicaciones en revistas científicas o actas de eventos sobre la temática, y al menos un año de experiencia en el área. Se excluyeron dos participantes que no alcanzaron la puntuación mínima definida, quienes no fueron incluidos en la base de especialistas para el cálculo del Índice de Validez de Contenido (IVC)^(16)^.

### Recolección de datos, instrumentos utilizados y variables del estudio

Los especialistas recibieron por correo electrónico un formulario dividido en tres secciones. En la primera, respondieron preguntas de autoevaluación mediante un sistema de puntuación para especialistas. En la segunda, validaron el contenido textual en cuanto a relevancia, claridad y pertinencia. En la tercera, se recopilaron datos demográficos.

### Tratamiento y análisis de los datos

El IVC es una medida de validez que determina la proporción o porcentaje de concordancia entre especialistas sobre un determinado contenido^(16)^. Se solicitó a los especialistas que calificaran la relevancia, claridad y pertinencia de cada contenido disponible en *Google Forms*. Los conceptos y las SV fueron clasificados mediante una escala tipo Likert con las siguientes opciones: 1 = Inadecuado; 2 = Parcialmente adecuado; 3 = Adecuado (requiere pequeños ajustes); 4 = Totalmente adecuado. Para calcular el IVC de cada contenido, se sumaron las respuestas 3 y 4 de los especialistas y el resultado se dividió por el número total de respuestas obtenidas para el ítem. Para su interpretación^(16)^, el valor mínimo aceptable del IVC debe ser 0,78. Los valores del IVC deben utilizarse para orientar decisiones sobre revisiones o rechazos del contenido evaluado. Si el IVC es bajo, puede indicar que el contenido no posee suficiente relevancia, claridad y pertinencia^(16)^.

## Aspectos éticos

Esta investigación se ajustó a las normas de la Resolución n.º 510/2016, que exime de evaluación por el Comité de Ética en Investigación cuando se trata de “[...] investigación cuyo objetivo es el desarrollo teórico de situaciones que emergen de forma espontánea y contingente en la práctica profesional, siempre que no se revelen datos que puedan identificar al sujeto [...]”, y a la Resolución n.º 674, del 6 de mayo de 2022, que refuerza dicho texto. Así, se envió a los especialistas el enlace del formulario, el cual incluía la invitación a validar el contenido y explicaba la exención de evaluación por parte del Comité de Ética. Los datos están archivados y bajo la responsabilidad de la investigadora, almacenados en una dirección electrónica y en una nube digital específicamente creadas para esta investigación, protegidas por una contraseña electrónica considerada segura por el proveedor de servicios de correo electrónico de la empresa *Google* ^®^.

## Resultados

El contenido del sitio *web* fue estructurado en torno al tema central de la adhesión y tres ejes: autoeficacia, apoyo social y CV, los cuales influyen en el desenlace de la adhesión al tratamiento antirretroviral. Para la traducción del contenido textual, se basó en la siguiente síntesis de evidencias: la autoeficacia^(8)^ presentó correlación significativa con la adhesión global mediante experiencias de molestia, discriminación, rechazo, inseguridad sobre cómo reaccionarían las personas cercanas al conocer el diagnóstico, nerviosismo, irritabilidad, ingesta de gran cantidad de comprimidos y otros efectos adversos causados por la medicación. Estas experiencias fueron traducidas como SV y articuladas con el apoyo social^(7)^ y el apoyo emocional, que mostró correlación significativa con los dominios de antecedentes de fallos de adhesión y la comunicación médico-paciente. También formaron parte de las SV los dominios de la CV^(9)^; entre aquellos asociados positivamente con la adhesión, se destacaron la función general, las preocupaciones relacionadas con la medicación y la confianza en el profesional de salud. El dominio que influyó negativamente en la adhesión fue la preocupación por el mantenimiento del secreto. En cuanto al ensayo clínico aleatorizado (ECA)^(10)^, se utilizaron los mensajes de texto basados en apoyo social para componer las orientaciones vinculadas a las SV.

El contenido del sitio *web* fue validado por 20 especialistas, de los cuales 15 (78,9%) eran mujeres; la edad promedio fue de 41,89 ± 10,954 años; 12 (57,9%) eran enfermeros de formación; 7 (31,6%) tenían maestría como el mayor nivel de titulación; y 13 (68,4%) trabajaban en el área asistencial (no exclusivamente). Entre los especialistas, 11 (55,6%) ejercían su profesión en el estado de Rio Grande do Sul, Brasil. La Tabla 1 presenta las variables continuas y el IVC, que alcanzó una puntuación >0,78 en todas las medias de relevancia, claridad y pertinencia. Por ello, el contenido del sitio *web* fue validado por especialistas en la temática y, por lo tanto, está apto para integrar la tecnología y avanzar hacia la siguiente etapa de evaluación con el público objetivo, es decir, personas adultas que viven con VIH.

**Tabla 1-:** Caracterización de la muestra de especialistas e Índice de Validez de Contenido según conceptos y situaciones de la vida (n = 20). Santa Maria, RS, Brasil, 2022

**Variable**	**Media**	**Mínimo**	**Máximo**	**Desviación-estándar**
Puntuación Modelo Fehring	6,26	4	13	2,621
Edad	41,89	27	60	10,954
Tiempo de Formación	16,89	2	38	11,140
Tiempo de Titulación	6,74	1	35	7,578
Tiempo de Experiencia Profesional	15,526	2	35	10,895
Índice de Validez de Contenido				
**Contenido**		**Relevancia**	**Claridad**	**Pertinencia**
**Conceptos**				
Adhesión		0,95	0,81	0,95
Autoeficacia		0,95	0,76	0,95
Apoyo social		0,95	0,67	0,86
Calidad de Vida		1,00	0,86	1,00
**Situaciones de la vida**				
1		0,95	0,90	0,95
2		0,95	0,95	0,95
3		0,90	0,90	0,90
4		0,95	0,95	0,95
5		1,00	1,00	1,00
6		1,00	0,90	1,00
7		1,00	0,95	1,00
8		0,90	0,76	0,86
9		0,90	0,86	0,95
10		0,90	0,90	0,90
11		0,81	0,81	0,90
**Media**		**0,94**	**0,87**	**0,94**

Los especialistas emitieron sugerencias que fueron analizadas por el equipo de investigación y, cuando pertinentes, incorporadas en la versión final del contenido. Esta etapa contó con la participación de un equipo calificado en lenguaje, gramática y comunicación. La redacción final de los conceptos, considerando el texto y las ilustraciones, se presenta en la Figura 2.

**Figura 2-:** Conceptos validados e ilustraciones finales del sitio *web*. Santa Maria, RS, Brasil, 2022

*VIH = Virus de la Inmunodeficiencia Humana

Además de los conceptos, el contenido textual validado (Figura 3) dio origen a las demás ilustraciones conforme a las SV.

**Figura 3-:** Representación de la articulación del contenido de las SV^†^ con los ejes estructurantes y el tema central – adhesión. Santa Maria, RS, Brasil, 2022

*VIH = Virus de la inmunodeficiencia humana; ^†^SV = Situación de la vida

Para la identidad visual del sitio *web*, se creó un logotipo con la palabra “conviva” (Figura 4), que expresa la idea de vivir y convivir con el VIH, así como la necesidad de adaptarse a una nueva rutina de vida. Para cada SV, se elaboraron ilustraciones que acompañan el contenido textual con el objetivo de involucrar al público objetivo y potenciar su comprensión. En la evaluación de los especialistas, el concepto de CV y la SV 10 utilizaron la misma ilustración por contener contenidos afines (Figura 4). El sitio *web* está disponible en el enlace: https://www.ufsm.br/pet/ciencia-da-computacao/conviva-1.

**Figura 4-:** Identidad visual e ilustraciones representativas de las Situaciones de la Vida. Santa Maria, RS, Brasil, 2022

*SV = Situación de la vida

## Discusión

El índice de concordancia entre los especialistas en la temática indicó que el contenido del sitio *web* presenta relevancia, claridad y pertinencia, siendo validado por dichos participantes para su uso con el público objetivo de adultos que viven con VIH. El contenido de las SV está articulado a los ejes autoeficacia, apoyo social y CV, relacionados con el desenlace de la adherencia^(7-9)^, concepto central de esta tecnología cuidado-educativa.

Los estudios que analizaron las barreras enfrentadas por adultos que viven con VIH para adherirse al tratamiento revelaron, según los resultados de un metaanálisis, que algunas barreras son de ámbito individual y otras están relacionadas con los servicios de salud^(17)^. En el ámbito individual se señalan con mayor frecuencia el olvido de la ingesta de los antirretrovirales y/o los efectos de los medicamentos^(18)^, el hecho de estar fuera de casa, en presencia de personas extrañas y el cambio en la rutina diaria^(19)^; además aparecían el sentimiento de estar bien (asintomático) o enfermo^(5)^, la depresión^(20)^ y el estigma^(21)^. Cabe destacar que tales barreras fueron propuestas y traducidas en este estudio y componen el contenido textual e imagético de las SV 1, 4, 5 y 6.

Existen otras barreras relacionadas con el lugar donde se realiza el tratamiento. Estas barreras son de índole programática; entre ellas se hallan la distancia geográfica al servicio de salud y la escasez de antirretrovirales^(17)^. Dichas barreras no fueron abordadas en esta propuesta debido a la complejidad asociada al sistema de salud y los distintos contextos locales.

Así, cuando se identifican tales barreras, es necesario proponer el fortalecimiento del vínculo con el servicio, buscar en la red de asistencia social de la persona apoyo e información con el fin de atenuar las implicaciones para la adherencia al tratamiento antirretroviral. El hecho de que el sitio *web* tenga la posibilidad de interferir en la retención de los usuarios a los servicios significa que esas personas pueden buscar ayuda en el equipo de salud. Un ejemplo se refiere a acciones junto al Servicio Social para viabilizar los derechos de esta población y las políticas públicas de salud relacionadas con el VIH^(22)^.

Todo lo expuesto indica que intervenciones combinadas podrían garantizar la supresión viral mediante niveles elevados de adherencia al tratamiento antirretroviral^(17)^. Además, la adherencia, como concepto central de la tecnología cuidado-educativa, se ha correlacionado con factores individuales como la edad avanzada, puntajes de resiliencia más elevados y la percepción de sentirse enfermo^(23)^.

Al articular tales evidencias con el concepto de autoeficacia para seguir el tratamiento^(19)^, se crearon y validaron el contenido textual e imagético de este eje, relacionados con siete SV. Como factor determinante para el tratamiento continuo, la percepción de autoeficacia permite que la persona que vive con VIH alcance buenos resultados de adherencia, sin presentar manifestaciones clínicas que, con el paso de los años, puedan interferir en su percepción de salud.

De la misma forma, el apoyo social, aunque es un factor significativo asociado a buenos resultados de adherencia, sigue siendo insuficiente para lograr el éxito, pues se debe considerar la superposición de factores que influyen en la adherencia^(24)^. Entre ellos, se destaca, en la dimensión del apoyo social, la comunicación entre la persona usuaria y el profesional de salud que la atiende, ya que se evidencia la correlación estadística con antecedentes de falla de adherencia^(7)^. Estas cuestiones están articuladas con las SV que involucran situaciones clínicas (SV 2, 3, 4, 6, 7, 9, 10 y 11), dado que, cuando el usuario final se identifique con preocupaciones relacionadas con situaciones de cuidado de su salud, se presupone que podrá acatar las orientaciones a partir de una comunicación adecuada entre usuario y profesional de salud. Ello se debe a que, entre los factores de pérdida de seguimiento del tratamiento antirretroviral, se encuentra la relación precaria entre el equipo de salud y las personas que viven con VIH^(2)^.

La articulación de los conceptos de adherencia y CV se presenta en cuatro SV (SV 8 a 11). Un estudio señala que existe un ciclo de retroalimentación entre la CV y la adherencia al tratamiento del VIH, de modo que, si la persona que vive con VIH obtiene buenos puntajes en la evaluación de la CV, podrá alcanzar buenos niveles de adherencia; del mismo modo, si dicha persona presenta una buena adherencia, tendrá una buena repercusión en su CV^(9)^.

Dado que la adherencia y la CV representan un ciclo, la evaluación de la CV indica que el entorno en el que vive la persona puede generar puntajes más bajos en este aspecto^(24)^. Esta cuestión está relacionada con la SV 1, en la cual la persona no revela el diagnóstico a desconocidos o conocidos a quienes no desea que sepan de su condición serológica, ya sea en el ámbito laboral o familiar. Esta SV también está contemplada y articulada con el concepto de autoeficacia.

De la misma manera, la dimensión de la CV que evalúa la salud psicológica mostró puntajes bajos en comparación con grupos de referencia^(25)^. Puntajes bajos en salud psicológica reducen la CV y la adherencia. Por lo tanto, la evaluación de la CV proporciona un cuidado adecuado en las dimensiones de salud física, psicológica, relaciones sociales, medio ambiente, nivel de independencia y espiritualidad de esta población.

Al comparar personas que aún no habían iniciado el tratamiento del VIH con antirretrovirales, quienes estaban en tratamiento relataron mejoras significativas en la salud física^(23)^, emocional, mental y en las actividades diarias^(26)^. Esta comparación confirma que el diagnóstico y el tratamiento precoces promueven la CV.

Los resultados de una revisión sistemática de estudios cualitativos, realizada en 2020 con el objetivo de explorar las experiencias y actitudes de personas que viven con VIH, señalaron el momento del diagnóstico como crucial debido a los sentimientos que se originan, tales como decepción, tristeza, miedo, desesperación, falta de conciencia y dolor. De este modo, los profesionales de la salud deben prepararse tanto para los momentos de vulnerabilidad como para las experiencias asociadas a distintos tipos de estigma: social, autoestigma y estigma de los profesionales de la salud. Por lo tanto, el apoyo social de estos profesionales es sumamente valorado y está vinculado a la mejora de la CV de estas personas^(27)^. La cuestión del estigma se aborda en las SV 1 y SV 5.

Aun en el ámbito del estigma, la CV de las personas que viven con VIH se ve impactada, particularmente en el dominio de la actividad sexual. La carga viral indetectable fue considerada transformadora en las trayectorias afectivas y sexuales de mujeres de 18 a 30 años que viven con VIH^(28)^. Al realizar entrevistas con jóvenes que viven con VIH/sida, un estudio brasileño realizado en Salvador (BA) demostró que esta población experimenta la necesidad de negociar el placer y la prevención y se posiciona como sujetos de “riesgo”. Al adoptar la noción de carga viral indetectable, se sienten seguras en la prevención y transforman los sentimientos de miedo, rechazo y la “posibilidad” de “peligro” para el otro (seronegativo)^(28)^.

Un estudio realizado en Suecia concluyó que la satisfacción sexual de mujeres que viven con VIH está relacionada con la CV^(29)^. En cuanto a las personas con pene, vivir con VIH puede desencadenar disfunción eréctil, perjudicando esa dimensión de la CV^(30)^.

A pesar de los avances en el ámbito biomédico, las cuestiones sociales relacionadas con el VIH, como el estigma y el prejuicio, siguen siendo impedimentos para las relaciones afectivo-sexuales. El estigma transforma el cuerpo de quien vive con VIH en un potencial transmisor del virus, un cuerpo que representa riesgo y sinónimo de promiscuidad. Además, el estigma y el prejuicio fomentan el sentimiento de culpa, presente de forma siempre activa o latente. La culpa surge por el hecho de que la persona se haya infectado o se haya expuesto al riesgo. Asimismo, persiste el sentimiento de responsabilidad no solo por su infección, sino también por la posibilidad de infectar a otra persona con la que llegue a relacionarse, incluso presentando buena adherencia y carga viral indetectable^(31)^. El tema de la sexualidad y sus nexos con los conceptos traducidos en este estudio para el público objetivo de adultos con VIH están representados en las SV 10 y 11.

En lo que respecta a la tipificación como una tecnología cuidado-educativa^(32)^ en la modalidad de sitio web informativo, se entiende que el uso de este recurso educativo atiende la necesidad de que los productos de innovación sean de bajo costo, de la misma forma que el envío de mensajes de texto por teléfono. Sin embargo, el envío de mensajes de texto se enfrenta a limitaciones tecnológicas en cuanto al envío de textos con muchos caracteres y la inserción de figuras. Cuando se envían imágenes junto con el mensaje de texto, se observa eficacia en la mejora de la adherencia y la satisfacción con los servicios^(33)^.

Con la posibilidad de acceder a internet mediante aplicaciones de navegación *web*, preinstaladas en los *smartphones* y conocidas como *browser* o *web browser*, es posible navegar por sitios o *webpages* muy similares a las aplicaciones nativas, tanto por su *layout* como por las funcionalidades que ofrecen. Las ventajas de acceder a tecnologías móviles directamente desde el navegador nativo del *smartphone* son: no requerir memoria interna para instalar múltiples aplicaciones, la posibilidad de acceder al contenido desde cualquier dispositivo y la alternancia entre tamaños de pantalla y sistemas operativos, sean móviles o no^(34-35)^.

Por último, el lenguaje textual e imagético de este producto contribuirá a las prácticas de literacidad en salud, partiendo del presupuesto de que las abstracciones, conceptualizaciones y comportamientos sociales y culturales son los que dan sentido al uso de las lenguas, incluidas aquellas difundidas en línea. Así, la producción de sentido mediante la elaboración de *layouts* para plataformas en línea pretende apoyar a los usuarios en la toma de decisiones y elecciones acertadas^(36)^ y responde a la necesidad de crear una identidad con el usuario final, generar interactividad y compromiso en el uso de la información.

Las limitaciones del estudio se refieren a la no inclusión de personas que viven con VIH en el proceso de creación del contenido de la tecnología cuidado-educativa en esta oportunidad. Para estudios futuros se recomienda el compromiso de dicha población objetivo.

En cuanto a las contribuciones del estudio, cuyo producto fue un sitio *web* informativo, cabe destacar el avance del conocimiento científico en el ámbito de la salud y la enfermería relacionado con la aplicación de la investigación en un modelo de traducción del conocimiento, y especialmente la disponibilidad de un producto cuya usabilidad está anclada en una tecnología cuidado-educativa. Esta modalidad puede contribuir a la autonomía de adultos que viven con VIH, proporcionándoles apoyo respecto a su condición multidimensional de salud y SV. Podrá cualificar la atención sanitaria al ser utilizada en los servicios de salud, con la posible mediación de profesionales asistenciales.

## Conclusión

El sitio *web* informativo creado pone a disposición conocimientos científicos que fueron traducidos y validados por especialistas en la temática en cuanto a su relevancia, claridad y pertinencia para ser utilizado por adultos que viven con VIH. La estructura del contenido textual y visual se ancla en conceptos fundamentales, tales como autoeficacia, apoyo social y CV, directamente relacionados con las experiencias de estos adultos. Estos conceptos desempeñan un papel crucial en la adherencia al tratamiento, que constituye el objetivo principal de esta tecnología cuidado-educativa.
